# Book Reviews

**Published:** 1871-08

**Authors:** 


### BIBLIOGRAPHICAL.

*Harris' Principles and Practice of Dentistry.* Tenth Revised Editon. By P. H. Austen, M. D., D. D. S., F. J. S. Gorgas, M. D., D. D. S., and T. S. Latimer, M. D., of the Faculty of the Baltimore College of Dental Surgery.

The great advances in Dental Physiology, Pathology, Surgery and Mechanism made it imperative that this first and oldest of Dental Text-Books should be in great part re-written, and rearranged, in order to bring it fully up to the present status of science, and preserve the reputation it has so long sustained throughout the civilized world. This has been done, and an edition of the work is now presented which we feel confident will be well received by the profession.

Many new and important illustrations have been added to the work, the contents of which are classified as follows: Part I. Dental Anatomy and Physiology. Part II. Dental Pathology and Therapeutics. Part III. Dental Surgery. Part IV. Dental Mechanics; with a chapter on Palatine Defects, prepared expressly for this edition by Norman W. Kingsley, D. D. S.

The enterprising publishers, Messrs. Lindsay & Blakiston of Philadelphia, have issued this new edition in a style creditable to themselves, it being printed on fine paper with large clear type, rendering it in every respect convenient for the dental student. The number of illustrations this new edition contains is four hundred and nine, an increase of nearly one hundred over the ninth edition of the work.

---

*Insanity in Women.* By Prof. H. R. Storer, M. D., Boston. The author of this interesting treatise ascribes a large proportion of the cases of insanity in women, being of a reflex nature, to diseases of the pelvic organs, and indicates the rational mode of treatment. He asserts that "the relative mortality of insane women is less than of insane men."

---

*Diseases of the Womb.* Uterine Catarrh frequently the cause of Sterility. New Treatment. By H. E. Gantillon, M.D. James Campbell, Publisher, Boston. This work is divided into: I. Anatomical and Physiological reflections on the Uterine Cavities. II. Causes of Uterine Catarrh. III. Symptoms of Uterine Catarrh. IV. Diagnosis of Uterine Catarrh. V. Prognosis. VI. Treatment. VII. Cases.

---

*The Virginia Clinical Record.*—A new monthly journal of medicine, surgery and the collateral sciences. Terms $1.00 per annum. W. W. Hazlewood, publisher, Richmond, Va.

---

*The Medical Cosmos.*—A new monthly abstract of medical science and art. By George J. Ziegler, M. D. Terms $1.00 per annum, 113 South 16th Street, Philadelphia, Pa.

---

*American Journal of Microscopy.*—Devoted to the elucidation of scientific and popular microscopy. By E. M. Hale, M.D. Editor. Published by G. Mead & Co., 182 S. Clark St., Chicago.

---

*The Medical World.*—A new monthly journal of American and Foreign medical, physiological, surgical, and chemical literature, criticism, and news. Edited by Reuben A. Vance, M.D. Wm. Baldwin & Co., Publishers, 21 Park Row, New York.

